# Hurdles in Basic Science Translation

**DOI:** 10.3389/fphar.2017.00478

**Published:** 2017-07-18

**Authors:** Christina J. Perry, Andrew J. Lawrence

**Affiliations:** ^1^Behavioural Neuroscience Division, The Florey Institute of Neuroscience and Mental Health, Parkville VIC, Australia; ^2^Florey Department of Neuroscience and Mental Health, University of Melbourne, Melbourne VIC, Australia

**Keywords:** knowledge translation, animal models, Clinical Trials as Topic, Reproducibility of Results, basic research

## Abstract

In the past century there have been incredible advances in the field of medical research, but what hinders translation of this knowledge into effective treatment for human disease? There is an increasing focus on the failure of many research breakthroughs to be translated through the clinical trial process and into medical practice. In this mini review, we will consider some of the reasons that findings in basic medical research fail to become translated through clinical trials and into basic medical practices. We focus in particular on the way that human disease is modeled, the understanding we have of how our targets behave *in vivo*, and also some of the issues surrounding reproducibility of basic research findings. We will also look at some of the ways that have been proposed for overcoming these issues. It appears that there needs to be a cultural shift in the way we fund, publish and recognize quality control in scientific research. Although this is a daunting proposition, we hope that with increasing awareness and focus on research translation and the hurdles that impede it, the field of medical research will continue to inform and improve medical practice across the world.

## Introduction

Globally, focus on the rate of knowledge translation is becoming more urgent. This is an issue that is relevant to researcher scientists, practitioners, government bodies and the general public alike. The cost of research and development for a successful drug is high, with estimates varying from $US 1 billion (Collins, 2011; Davidson, 2011) to United States $2.6 billion (DiMasi et al., 2016; Langley et al., 2017). Furthermore, it typically takes 10–20 years to bring a new treatment from initial discovery to final approval. Although there are dissenting views as to the exact figures, and how they are derived – particularly where it concerns cost (Avorn, 2015), there is no doubt that it takes significant time and resources to engineer and implement medical advances. A large portion of this cost can be attributed to drugs that fail at different stages of the clinical trial process (DiMasi et al., 2016).

In Australia, the National Health and Medical Research Council (NHMRC) instituted a Translational Research Faculty which opened in 2015. This is one of many such institutions that are appearing around the world. As part of this drive, a number of key proposals were made to the NHMRC, which identified and localized problems with knowledge translation in specific fields. These proposals span a wide range of fields, and identify obstacles at a number of different stages of the knowledge translation process. For example, we still do not have any effective treatment to modify the course of Alzheimer’s disease despite over 30 years of research, reflecting obstacles at the level of basic research, and the translation of basic findings to clinical trials (Brodaty et al., 2015). In the field of oncology, there are validated cancer treatments, which are improving all the time, but they are extremely expensive, and frequently people are not getting the treatment they need. This is particularly true for those in lower socioeconomic groups and remote areas, and indicates a problem with translation from clinic to practice (Roder et al., 2015). In the area of falls and fracture prevention there is a problem of a persistent evidence-practice gap. Here medical professionals are not necessarily making recommendations based on research. For example, despite resounding evidence that bone density can be improved by a regime of exercise that combines strengthening and balance, health professionals are not always recommending these practices to older and at-risk individuals. This may be in part due to a lack of awareness at the clinical level regarding the specific advantages of this type of regime, but also due to lack of government funding for specific programs aimed at older individuals (Buchbinder et al., 2015). In the case of type II diabetes, the cause is well established, and most if not all clinicians and practitioners are aware of it, and yet some of the preventative measures are still not getting across to the public. This is probably due to compliance with interventions being difficult to maintain, particularly as regards lifestyle interventions such as dietary modification (Colagiuri and Johnson, 2015). On the other hand, for asthma, there are effective treatments, and compliance is good. However, now there are new problems emerging, such as over-diagnosis, over-treatment, few new therapies being developed. In other words, the problem here is the lack of a new approach, and for this to occur practical knowledge needs to be effectively translated back to the researchers (Gibson et al., 2015).

From the above it is clear that there are several stages in the translation process (Davidson, 2011). First there is a need to translate basic science to human research, and then take findings from clinical studies to improving public health. This may involve translating data from clinical trials to health recommendation and guidelines, and then the adoption of these guidelines into evidence-based practice. From there, medical advances need adoption and compliance from consumers. Finally, it is important that knowledge regarding efficacy, reception and other relevant clinical observations is translated back to researchers to ensure that they are working on the pertinent problems.

Put simply, the issue of knowledge translation is one that requires multidisciplinary attention and awareness from all levels and stages of the health and medical field (from basic scientists to industry, to regulatory bodies, to practitioners and consumers). In this commentary, as basic scientists, we will focus on the transition from basic research to clinical trials. This is not to ignore the fact that there are many other factors – economic, political and cultural – that influence the gap between evidence and practice. To address all of these, however, is beyond the scope of this review. The question that we consider is why so many new discoveries fail to be translated into new practices. In this context we examine three important factors that may contribute to the slow development of new and effective treatments: the way disease is modeled during basic research, the complex nature of the targets that are being examined, and the issue of reproducibility (or lack thereof) in research findings. Our hope is that by identifying weaknesses in we will raise awareness about steps we can take to improve research practices. This process of revaluation, validation and adjustment is fundamental to science as a discipline, and important for ensuring that our field remains reliable and relevant.

## The Importance of A Good Model

Following target discovery, as much as 90% of novel medications fail to make their way through the different phases of clinical trial to approval. The majority fail to pass at phase II – the transition through proof of concept to large scale trial at Phase III (Cook et al., 2014; Perrin, 2014; DiMasi et al., 2016). Many critics attribute this high rate of attrition to the models on which the targets are discovered – usually laboratory rodents; the argument being that the differences between animal models and human disease are too great to be able to draw valid conclusions between them. In other words, targets derived from animal models are not suitable for use in clinical populations, and hence fail at trial. One highly cited example is the random relationship between genomic changes in response to immune stressors in humans compared to mice (Seok et al., 2013). Certainly, this particular finding is controversial (e.g., Takao and Miyakawa, 2015), however, it has incited much debate as to the applicability of findings derived in mouse models for developing treatments for human disease. Thus, our first question regards research using animal models: Are they relevant and informative? Can they be improved and if so, how? Are there available alternatives?

Indeed, there are many new and exciting models being developed, for example human pluripotent stem cells, 3D culture systems, genome editing *in vitro*, and good quality normal and diseased human tissue, derived from post-mortem or live biopsies (Langley et al., 2017). Importantly, however, there is as yet no good behavioral component in alternative (non-animal) models, and behavior is a vital component of many diseases. Therefore, there is still a critical place for non-human animal models in medical research; however, the model(s) should be validated.

Much current medical research is heavily dependent on the use of transgenic mouse models of disease. These models have provided us with a vast amount of important information regarding a range of different human disease. However, the phenotypes of transgenic models do not necessarily induce the relevant disease markers. For example, an investigation into the mutant mouse overexpressing human TDP-43 gene, a model of amyotrophic lateral sclerosis (ALS), revealed that morbidity in these animals arose from reduced gut motility, rather than ALS symptoms (Hatzipetros et al., 2014). In addition, phenotype may be affected by issues such as husbandry conditions, and the wild-type strain the mutant is crossed with, genetic drift and genetic contamination within an in-house colony, and even the specific pathogen status of the facility (Justice and Dhillon, 2016). It is therefore critical that the model is validated regularly to ensure that the phenotype expresses the disease biomarkers of interest, in order to avoid confounds arising from these artifacts.

It is also important to consider that many diseases emerge later in life, or after prolonged and chronic exposure to particular conditions. To recreate biomarkers of these diseases, whether in outbred, inbred, or transgenic subjects, requires time, patience and long term commitment to individual experiments. Substance use disorders are an example of a disease that is typically developed over a long period of time. People who present for treatment for substance use have usually consumed large quantities of drug over extended periods of time. However, many animal models of substance use provide subjects with only a short period of time with access to the drug of abuse. This may mean that the necessary biomarkers do not emerge. Indeed, length of alcohol exposure can affect the efficacy of target treatment (Patkar et al., 2016), so targets that are derived from short-term experiments may not necessarily be translatable to the disease itself. Clearly, it is important that we establish prior to experimentation that known biomarkers in the human disease are present in the animal model of choice. This might be done using post-mortem tissue, or other clinical observations, allowing clinicians and researchers to correlate markers of human disease with those seen in the relevant model. As such, it clearly requires open communication from the clinical level back to basic scientists.

An elegant solution to ensuring the availability and regular validation of good animal models would be to make this type of work a community effort. Validating models is time consuming and expensive, like any research. Furthermore it is difficult to publish; so many smaller laboratories simply cannot afford such investment alongside innovative research. As such, the burden of model validation could be shared across laboratories (Perrin, 2014). This could take the form of a public repository, such as the Alzheimer’s disease neuroimaging initiative^1^. This project provides members with access to a collection of data that includes cognitive tests, CSF and blood biomarkers that can be used to predict the disease. Another possibility would be to have journals that are dedicated to publishing model development and validation studies. Along these lines, reviews that describe animal models around particular conditions are emerging (Hainsworth et al., 2017; John and Said, 2017), and may be useful for weighing up the quality of model available, as well as for designing research strategies for testing new therapeutics. In fact, such research has the potential to be highly cited, given that other researchers studying a particular disease would refer to it as justification for their own methods. Alternatively, specific funding could be provided for characterisation projects, providing both incentive and means to carry out this sort of work (Perrin, 2014). Providing funding and opportunity for publication and recognition in turn gives incentive for laboratories to engage in proper development, characterisation and validation, and should therefore mean that the models that exist are more robust.

Re-deriving and regular validation of animal models improves the quality of basic research – and indeed in some of the examples cites, such as the TDP-43 gene, new and improved animal models have been developed (Alfieri et al., 2014, 2016). However, improved recognition for this sort of work is essential to ensure that it occurs regularly. It is also worth noting that no one animal model is likely to cover all aspects of complex human disease (Hainsworth et al., 2017), meaning that novel therapeutics should be considered within a range of preparations/systems. There will also always be certain elements of human experience – especially cognition and affective state – that are extremely difficult to model in laboratory animals. While this caveat should not be forgotten, it nevertheless does not detract from the value of a robust animal model for understanding the neurobiology and pharmacology of both maladaptive and healthy human conditions.

## Understanding your Target

### Biased Signaling

New pharmaceutics frequently fail clinical trials because they have contraindications and off-target effects that make them unsuitable for general medical practice. Interestingly, it has become evident that these undesirable consequences of drug administration may arise through different downstream signaling pathways from that which mediates the target drug effect. For example, mu-opioid receptor agonists such as morphine are powerful analgesic agents, but also are highly addictive, and can cause potentially fatal respiratory depression. The analgesic properties arise where ligand binding recruits G_i_ protein, activating inwardly rectifying potassium channels and inhibiting adenylyl cyclase; while drug tolerance and respiratory depression occur when the ligand recruits β-arrestin (although as yet no mechanism for this effect has been described) (Raehal et al., 2005). There has been a protracted search for agents that could replace morphine, with its potentially lethal side effects, however, the vast majority have been rejected at trial simply because there was no way to control for which of these pathways were selected (Manglik et al., 2016).

However, since crystal structures of the opioid receptors have now been described, emerging technology now allows for structure-based discovery of drugs targeting this family (Marino et al., 2017). We also now know that certain ligands will preferentially recruit G-protein signaling over β-arrestin, and vice versa (Rajagopal et al., 2010). The opioid receptor is one example, but this is also occurring with other families, for example with drugs that target muscarinic acetylcholine receptors (Bradley et al., 2016). Once verified for their specificity, for example in appropriate knockout animals, such biased ligands can be carefully selected based on which downstream cascades or pathways are activated. Accordingly, researchers and drug discovery groups will be able to engineer new agents that are much more selective (Manglik et al., 2016).

Intuitively, this new approach would seem to have the potential to streamline preclinical drug discovery, and reduce the cost and time involved with clinical trials. Currently it is a novel technology, and although it improves predictions regarding how a drug will perform *in vivo* from its properties *in vitro* (Luttrell et al., 2017), there is as yet no evidence regarding improvements to success rates clinically. These are, however, relatively early days in this regard and the future may see this principle translate.

### Heteromerization

During the drug discovery process, it is also very important to know how the target receptors behave *in vivo*. Receptor subunits do not necessarily gather in homomeric formation within the cell membrane. Indeed receptors may be heterodimers or even larger mosaics made up of different receptor type subunits (Fuxe et al., 2014). Thus a compound that is selective for a certain homomeric receptor may be less effective *in vivo*, where the receptor may exist in a heteromeric formation.

The metabotropic glutamate 5 (mGlu5) and adenosine A2A receptors are one example of this phenomenon. mGlu5 receptors have been investigated as targets for drugs to treat depression, fragile X, cognitive decline and dementia, and addiction (Swanson et al., 2005; Bird and Lawrence, 2009; Olive, 2010). Some of these drugs, such as Fenobam – an anxiolytic/antidepressant, and negative allosteric modulator of mGlu5 – are FDA approved, however, many have failed at clinical trial, and none are broadly prescribed (Marino and Conn, 2006). Notably, however, the mGlu5 and A2A receptor show close co-localisation in the membranes of human embryonic kidney cells and co-precipitate from rat striatal tissues (Ferre et al., 2002). Functionally, this is evident in the synergistic effect of compounds that target these two receptor types. Using both genetic and pharmacological antagonism of A2A receptor and mGlu5 receptors, greater than additive effects have been observed on addiction-related behavior for alcohol (Adams et al., 2008), cocaine (Brown et al., 2012), and methamphetamine (Wright et al., 2016).

Again, this is not an isolated case. Similar interactions have been observed with Galanin and Neuropeptide Y for treating emotional, metabolic and cardiovascular treatments (Diaz-Cabiale et al., 2014), and A2A receptors with dopamine D2 receptors for treating Parkinson’s (Beggiato et al., 2014). This is relevant since in preclinical research, therapeutic agents being investigated may target specific receptors, and are tested one at a time, with the assumption that the receptors exist in homomeric form *in vivo*. As such, the doses required for behavioral effect may be higher, and, should receptor formation be different in human tissue compared with the tissue of laboratory animal, or in diseased tissue compared to normal tissue, then a drug that is effective in the lab may prove to be less so (or more so) in clinical trial. On the other hand, knowledge of receptor structure – whether homomeric or heteromeric – particularly how they form in human tissue, and in brain regions known to subserve the target behavior, should furnish us with the framework to design drugs that target heteromeric complexes or formulate specific combinations of drugs that may have much more sensitive effects. Understanding the full effects of the compounds being used is also important. In other words, just as we should be regularly validating our models to ensure that they are faithfully reflecting the disease of interest, we should also have ready access to accurate and up-to-date information about the ligands we are using, and how our drug targets exist within the relevant human disease. As with animal models, public-access databases that provide information about all known effects of new compounds together with links to relevant publications may provide more reliable and complete data for understanding drug effects. These do exist – for example the IUPHAR/BPS guide to pharmacology^2^, but should be curated regularly in order to remain valid. This is something that requires funding, and hence needs to be addressed at a policy level.

## Reproducible Results

Reproducibility of basic research findings is another contentious issue, and one which may contribute to high rates of failure in clinical trials. In 2012, researchers from Amgen biotechnology reported on a decade-long attempt to replicate key findings in the field of hematology and oncology. Out of the 53 landmark studies targeted, only 6 could be confirmed (Begley and Ellis, 2012). Although this is a particularly low figure, this trend is not an isolated finding (Prinz et al., 2011), nor is it unique to the field of oncology or research carried out by industry (Nosek et al., 2015). Indeed, increasing awareness of this issue has led to projects such as the Reproducibility Project (Baker and Dolgin, 2017), and the Transparency and Openness Project (Nosek et al., 2015) which probe the reproducibility of influential discoveries. It should be noted, though, that a single failure to replicate may not necessarily indicate lack of reproducibility. Indeed, in another project where many labs, rather than just the one, attempted to replicate each finding (here key findings in the field of psychology), 10 out of 13 key findings could be reliably reproduced within a 95% confidence interval (Klein et al., 2014). Although this provides us with a much less pessimistic outlook on the value and rigor of contemporary basic research, it nevertheless emphasizes the value of making public attempts – whether successful or not – to replicate important findings in the literature.

One of the points that was particularly concerning in Amgen’s study is that many of the findings that could not be reproduced had formed the foundation of ongoing programs of research without any initial attempt to replicate the original finding – suggesting that there was a great deal of ongoing research based on somewhat “loose” foundations (Begley and Ellis, 2012). This may be because reproducing already published and accepted data is regarded as a waste of time and resources, both of which are valuable and scarce. In a highly competitive environment where publication on innovation is key to career success, pursuing experiments that have already been accepted may not be a key priority. Compounding this is a documented difficulty in publishing results which fail to replicate previous published results (Menger and Haim, 1992; Dirnagl and Lauritzen, 2010). The consequent financial impact should irreproducible targets make their way into the clinical trial phase, however, can be profound. Clinical trials are expensive, and it is greatly preferable that an ineffective target be dismissed at the preclinical stage.

This reproducibility is due at least in part to an ingrained publication bias. As mentioned, there is strong incentive to publish novel and innovative findings rather than research that confirms the findings of others, as well as a disincentive to challenge prominent theories. There is also an increasing push for quantity rather than quality within findings. This can be partly attributed to a business-oriented culture found in research carried out by pharmaceutical companies (Cook et al., 2014); however, the problem is clearly not isolated to research within the private sector. Much of the academic world works under a mantra of “publish or perish” which places undue pressure on researchers to publish novel findings as quickly as possible without subjecting them to as much rigorous examination as they perhaps deserve, as well as discouraging attempts to confirm/replicate findings already published as discussed above. So much career development and grant funding is dependent on a track record that illustrates a high research output (i.e., many publications). It is clear that to improve the quality and hence rate of translation of basic medical research there needs to be a cultural change such that published findings are more open to validation, and that greater recognition is given to those that seek to validate and verify research methods. On this note, it is worth remembering that certain government-issued policies regarding care and use of animals for scientific process explicitly state that animals should not be used simply to repeat experiments within a project (National Health and Medical Research Council, 2013). Although this is done to advance the principle of reduction in animal use, this can also unwittingly impact efforts to replicate findings. Therefore, these issues should be considered carefully when drafting and implementing research policy.

Non-reproducible results can arise from various factors, including sampling bias. However, one notable and avoidable source is through errors in experimental design or data analysis (Settleman and Cohen, 2016). This can in fact be overlooked during the peer review process, as expertise in the biomedical field does not necessarily come with a high level of expertise in statistics and data analysis. Journal editors may benefit from enlisting expert consultants in addition to peer review. A statistician, for example, could provide expert review on data analysis and experimental design, while a technical scientist might comment on the methods and interpretations drawn from the findings (Settleman and Cohen, 2016). This would ensure that published research adheres to more rigorous statistical and experimental design (specific experts may pick up design flaws that are overlooked by other peers). So it seems that there is a call for a change in the culture of scientific publication – in the way we review new manuscripts, and the value placed on publication of null results and attempted replication (failed or successful). In this regard, some journals are in fact working to ensure that papers submitted do adhere to a more rigorous standard in terms of experimental design and data analysis (Curtis et al., 2015). This is encouraging, as it indicates that the problem of consistency and clarity in design and analysis is being acknowledged. However, it is important that these standards are upheld and rigorously reviewed.

Systematic reviews can also be very useful in reducing the problem of reproducibility. In theory, these should generate global evidence surrounding a particular issue by bringing together research on one problem from many and far reaching sources (Grimshaw et al., 2012). As such they help to understand the extent to which results can be generalized to a clinical population, how likely they are to be reproducible. Undertakings such as Cochrane reviews are invaluable in this way. These are extremely laborious undertakings, but often become important resources when it comes to translating basic research. However, they are still reliant on published findings, and do not get around the difficulty apparent in publishing data that fails to reproduce previously published data (Palma and Delgado-Rodriguez, 2005; Dirnagl and Lauritzen, 2010).

## Summary and Solutions

When considering the time lag of knowledge translation, it is important to remember that slow and steady research is not necessarily a bad thing (Hanney et al., 2015). It is important to ensure that high quality and reproducible data are being generated in basic biomedical research, and that the targets identified are rigorously tested in the clinic. Naturally, this is a time-consuming process, and generation of ineffective targets at times is going to be unavoidable. Nevertheless, it is better (and cheaper) that potential targets be discarded at the preclinical level should they prove ineffective. Here we have identified three ways of facilitating this process: (1) having access to high quality and well validated models for designing and testing novel treatments at a preclinical level, and ensuring that these models express the relevant disease biomarkers (2) having a good understanding of how your target behaves in human tissue, and designing your treatment accordingly, and (3) ensuring that published research can be reproduced or refuted so that knowledge being translated is accurate. Potential solutions to these issues involve providing direct support and recognition for work that targets these issues. For example, dedicated project funding for work that seeks to validate models, or reproduce previously published findings will help encourage researchers to engage in this type of work. Furthermore, changes to the culture of peer review publication which permits more rigorous validation of scientific methods, as well as publication of results that refute accepted findings will help to overcome the issue of reproducibility.

Some of this change is already underway. As discussed, there are repositories for information regarding specific disease models. The literature is beginning to include discussion around the merits and pitfalls of different animal models (Hainsworth et al., 2017; John and Said, 2017). There are projects in place that aim to reproduce the major findings in the field (Nosek et al., 2015), some journals are putting in place a negative results section, where failure to reproduce can be recognized (Dirnagl and Lauritzen, 2010) as well as implementing higher standards for experimental design and data analysis (Curtis et al., 2015). It is vital that we continue these efforts in order to ensure that the field of biomedical research remains relevant.

## Author Contributions

Both CP and AL contributed to conceptualizing, writing and editing the manuscript.

## Conflict of Interest Statement

The authors declare that the research was conducted in the absence of any commercial or financial relationships that could be construed as a potential conflict of interest.

## Footnotes

1 http://adni.loni.usc.edu

2 www.guidetopharmacology.org
